# School Bag Weight as a Barrier to Active Transport to School among New Zealand Adolescents

**DOI:** 10.3390/children5100129

**Published:** 2018-09-20

**Authors:** Sandra Mandic, Roman Keller, Enrique García Bengoechea, Antoni Moore, Kirsten J. Coppell

**Affiliations:** 1 Active Living Laboratory, School of Physical Education, Sport and Exercise Sciences, University of Otago, PO Box 56, Dunedin 9054, New Zealand; kellerro@student.ethz.ch; 2 Department of Medicine, Dunedin School of Medicine, University of Otago, PO Box 56, Dunedin 9054, New Zealand; kirsten.coppell@otago.ac.nz; 3 Department of Physical Education and Sport Sciences, Faculty of Education and Health Sciences, University of Limerick, V94 T9PX Limerick, Ireland; enrique.garcia@ul.ie; 4 School of Surveying, University of Otago, PO Box 56, Dunedin 9054, New Zealand; tony.moore@otago.ac.nz

**Keywords:** Active transport, school, school bag, adolescents, parents

## Abstract

Background: Excessive school bag weight is a barrier to active transport to school (ATS). This study examined parents’ and adolescents’ perceptions of school bag weights and actual school bag weights for adolescents in New Zealand. Methods: Parents (n = 331; 76.7% women) completed a survey. Adolescents (n = 682; age 15.1 ± 1.4 years; 57.3% boys) completed a survey, underwent anthropometry, and had their school bags weighed. Results: Overall, 68.3% of parents perceived that adolescents’ school bags were too heavy to carry to school. This parental perception differed by adolescents’ mode of transport to school (active/motorized/combined: 35.1%/78.4%/68.8%, *p* < 0.001). Adolescents perceived that their school bags were too heavy to carry to walk (57.8%) or cycle (65.8%) to school. Adolescent perceptions differed by mode of transport to school (for walking (active/motorized/combined): 30.9%/69.2%/55.9% agree, *p* < 0.001; for cycling: 47.9%/72.8%/67.7%; *p* < 0.001). Actual school bag weight was, on average, 5.6 ± 2.1 kg. Relative school bag weight (% of body weight) was higher for boys and underweight adolescents compared to their counterparts. Neither absolute nor relative school bag weight differed by mode of transport to school. Conclusions: School bag weight was perceived a barrier to ATS and was a greater perceived barrier among users of motorized versus active transport. Perceptions of school bag weights should be considered in future ATS interventions.

## 1. Introduction

Excess school bag weight and associated factors, including school bag weight relative to child/adolescent weight, method of carrying, and time spent carrying a school bag are thought to be key factors responsible for musculoskeletal complaints among children and adolescents [1,2]. Musculoskeletal complaints are a common issue in school-aged children and adolescents and have been identified as an important public health problem [3,4]. Relative school bag weight is associated with neck and shoulder pain in adolescents [2]. The musculoskeletal implications of school bag weight for adolescents may differ depending on the mode of transport to school [2]. For example, a greater proportion of Iranian adolescents who walked to school reported neck pain compared to those who traveled by car or bus [2]. However, a recent systematic review did not find evidence that school bag use increased the risk of back pain in children and adolescents [5].

The average weight of adolescents’ school bags varies across countries, ranging from 2.8 kg to 6.6 kg [2,6,7,8]. Previous studies related to proposed school bag weight recommendations primarily focused on musculoskeletal issues (back, shoulder, and neck pain) among adolescents [9,10,11]. Other important considerations include physical inactivity and a sedentary lifestyle, which contribute to poor health, including obesity and reduced psychosocial health [12]. Given the decline in physical activity (PA) from childhood to adolescence [13], with low proportions of adolescents achieving recommended daily PA levels [14,15], encouraging active transport to school (ATS) could be one intervention to increase PA in this age group. ATS is a convenient way to integrate PA into everyday life, and to maintain or increase PA levels in adolescents [16,17]. In the United Kingdom, teenage girls are six to eight times more likely to meet PA recommendations if they use ATS [18,19]. Although the prevalence of ATS varies among countries [20,21], ATS rates among adolescents have consistently declined over the last decade in developed countries [22], including New Zealand [23]. In 2010–2014, only 28% of New Zealand adolescents regularly walked and 3% cycled to school [23].

Common barriers to ATS for adolescents include distance to school, time constraints, lack of social support, convenience of being driven to school, weather, environmental factors, and traffic safety concerns of both adolescents and their parents [24,25,26]. Actual or perceived excess school bag weight may be another modifiable barrier to ATS for adolescents [26]. Few previous studies have examined children’s perception of their school bag weight [27,28] and the relationship between school bag characteristics and mode of transport to school [1,2]. However, those studies did not explore the relationship between adolescents’ and parents’ perceptions of school bag weight and mode of transport to school. Therefore, this study examined parents’ and adolescents’ perceptions of school bag weight and measured actual weights of school bags of adolescents from Dunedin, New Zealand. To our knowledge, this is the first study to examine the implications of school bag weight as a barrier to adolescents’ walking and cycling to school.

## 2. Materials and Methods

Data were collected as a part of the Built Environment and Active Transport to School (BEATS) Study conducted in Dunedin, New Zealand, between 2014 and 2017 [29,30]. All 12 secondary schools in Dunedin participated in this study. Recruitment of study participants and assessments are described in detail elsewhere [30]. In brief, parents of adolescents were recruited through schools, workplaces, social media, and at adolescent sporting events during 2014–2017. Parent participants signed their consent either on paper or online. Adolescents were recruited through their schools in 2014–2015. Written consent was obtained from all participating adolescents. For adolescents under 16 years of age, parents signed either parental opt-in or opt-out consent based on the school’s preference. The study was approved by the University of Otago Human Ethics Committee (reference number: 13/203).

### 2.1. Participants

A total of 365 parents completed an online or paper survey. After excluding parents who lived at the same address (n = 9) and/or did not have relevant survey data (n = 25), 331 parents were included in the analysis. A total of 1780 adolescents completed an online survey, and after exclusions, 682 adolescents were included in the analysis. Those excluded were adolescents who had invalid surveys (n = 38), incomplete student and/or parental consent (n = 79), no school bag weight measured (n = 365), declined anthropometry (n = 35), partial school bag weight at the time of measurement (n = 475), missing data on whether their school bag was full or partially full (n = 51), or who boarded at school (n = 55). 

### 2.2. Assessments

Parent participants completed the study questionnaire. Adolescents completed an online questionnaire, underwent anthropometry assessment, and had their school bags weighed on the same day.

#### 2.2.1. Questionnaire

Parents and adolescents completed the BEATS Study Parental or Student Survey, respectively. Parents had the option to complete their 20- to 25-min survey either online or on paper in their own time. Adolescents completed a 30- to 40-min online questionnaire at school under the supervision of research staff. Details on student and parent questionnaires have been described elsewhere [30]. Survey questions were related to demographics (age, gender, ethnicity), travel to school behaviors, and perceptions of walking and cycling to school. Home and school addresses were geocoded (converted into coordinates), then used to calculate distance to school using the shortest path on a connected street network using Geographic Information System (GIS) network analysis [30]. To determine the New Zealand index of deprivation (a measure of neighborhood socioeconomic status, with 1 = least deprived to 10 = most deprived), the geocoded addresses were matched with index values provided by the New Zealand Index of Deprivation Study [31], reported by census meshblock. Usual transport to school was assessed for each transport mode individually, as described previously [26]. Based on main modes of transport to school and multimodal transport, adolescents were classified into active transport users (walking, cycling, or riding a nonmotorized scooter “most/all of the time”), motorized transport users (taking the bus, driving, or being driven to school), or combined motorized and active transport users. Adolescents’ perceptions of school bag weight as a barrier to ATS were assessed using 2 separate items for walking and cycling to school (“I have too much to carry to walk/cycle to school”), whereas parental perceptions were assessed using a single item (“My child has too much to carry to walk or cycle to school”). These items were assessed using a 4-point Likert scale anchored in “strongly disagree” (1) and “strongly agree” (4). To calculate the proportion of participants agreeing with each statement, 4-point Likert scale items were recoded as “disagree” and “agree.”

#### 2.2.2. Adolescent Anthropometry Measurements

Height (custom-built portable stadiometer), weight (A&D scale UC321, A&D Medical, San Jose, CA, USA), and waist circumference (metal measuring tape) were measured using standard procedures, as described previously [14,30]. Height and weight were measured in school uniforms (without shoes, sweater, and jacket). Weight was recorded to the nearest 0.01 kg and reduced by 0.5 kg to account for clothing [14]. Weight status category was determined using international age- and sex-specific cutoff points with body mass index (BMI; weight divided by height squared (kg·m^−2^)), with BMI < 17 kg·m^−2^ indicating underweight [32].

#### 2.2.3. School Bag Weight Measurements

Adolescents’ school bags were weighed using a digital electronic scale (A&D scale UC321, A&D Medical, San Jose, CA, USA) and recorded to the nearest 0.01 kg. The measurement was performed by research staff at the time of the survey. Adolescents were asked at the time of measurement to report whether their bag was full weight (contained everything they brought to school that day) or partial weight. Only adolescents with full school bag weight measurements and available anthropometry data were included in this analysis.

### 2.3. Data Analysis

Demographic characteristics were analyzed using descriptive statistics. Differences between the groups were compared using *t*-test and chi-square test for continuous and categorical variables, respectively. Bivariate correlations were examined using Pearson’s product moment correlations. Data are reported as frequencies (percentage) for categorical variables and mean ± standard deviation for continuous variables. A *p*-value < 0.05 was considered statistically significant. Analysis was performed using SPSS software version 24.0.

## 3. Results

### 3.1. Parental Characteristics

Overall, parent participants (average age: 47.4 ± 5.1 years; Table 1) were predominantly women (76.7%) and married (72.8% of the total population), and approximately half of the participants had a university degree (55.3%) and worked full time (55.0%). Adolescents of participating parents were, on average, 14.9 ± 1.6 years of age (48% boys) with almost three-quarters travelling to school using motorized transport only (72.8%), followed by active transport (22.4%) and combined active and motorized transport (4.8%).

### 3.2. Parental Perceptions

**Figure 1:**
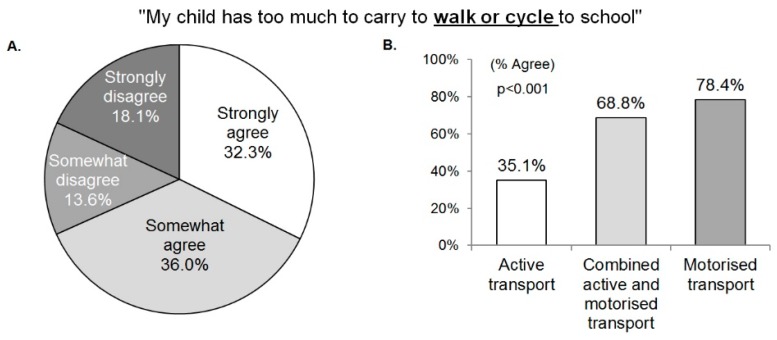
Parental agreement with the statement “My child has too much to carry to walk or cycle to school”: (**A**) in a total sample, and (**B**) by adolescents’ mode of transport to school.

Overall, 68.3% of parents perceived that their adolescents’ school bags were too heavy to walk or cycle to school (Figure 1A). This perception differed significantly by adolescents’ mode of transport to school (Figure 1B). Parental perception that adolescents’ school bags were too heavy to walk/cycle to school was negatively associated with the frequency of adolescents walking to school in the previous week (r = −0.35) and positively correlated with the frequency of adolescents being driven to and from school (r = 0.29 and r = 0.28, respectively; all *p* < 0.001), even after accounting for distance from home to school. Parental perceptions did not significantly differ by adolescent age (agree: 14.8 ± 1.5; disagree: 14.9 ± 1.6 years; *p* = 0.535) or gender (63.5% for boys; 72.7% for girls; *p* = 0.074).

### 3.3. Adolescents’ Characteristics

Adolescent participants (n = 682; average age: 15.1 ± 1.4 years; 57.3% boys) (Table 1) lived, on average, 6.1 ± 6.9 km from school. Overall, 4.5% of adolescents were underweight, 69.1% healthy weight, 19.7% overweight, and 6.7% obese. Most adolescents travelled to school using motorized transport only (60.5%), followed by active transport (24.5%) and combined active and motorized transport (15.0%). Participating girls were, on average, younger than boys (14.9 ± 1.5 versus 15.3 ± 1.4 years; *p* < 0.001). No significant gender differences were observed for ethnicity, distance to school, travel to school behaviors, or weight status.

### 3.4. Adolescents’ Perceptions

**Figure 2:**
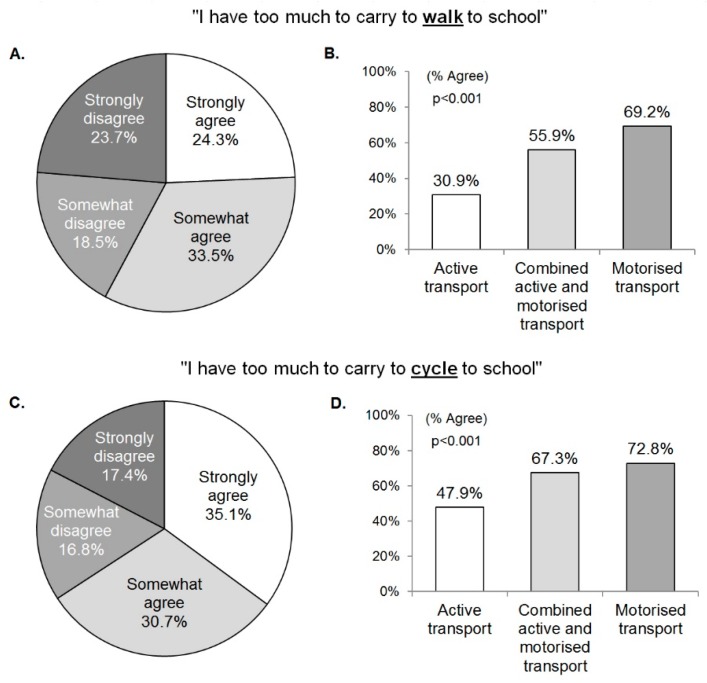
Adolescents’ agreement with the statement that they have too much to carry to (**A**,**B**) walk or (**C**,**D**) cycle to school (**A**,**C**) in a total sample and (**B**,**D**) by mode of transport to school.

**Figure 3:**
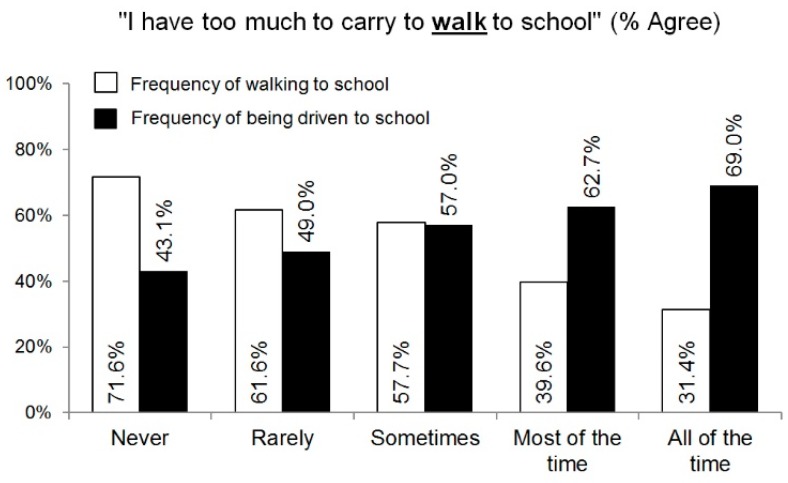
Adolescents’ agreement with the statement that they carry too much to walk to school by frequency of walking and being driven to school.

More than half of the adolescents perceived that their school bags were too heavy to walk (57.8%) or cycle (65.8%) to school (Figure 2A,C). Adolescent perceptions of carrying too much to school were not significantly different by age (agree: 15.1 ± 1.5; disagree: 15.1 ± 1.4 years; *p* = 0.867). However, they differed by gender, weight status, and mode of transport to school. A higher proportion of girls than boys agreed with each of the statements (for walking: girls: 64.0% versus boys: 53.2%, *p* = 0.005; for cycling: girls: 73.4% versus boys: 60.1%; *p* < 0.001). Compared to underweight and healthy-weight adolescents, a higher proportion of overweight and obese adolescents agreed that they their school bag was too heavy to walk to school (64.2% versus 54.8%; *p* = 0.024), whereas no difference between the groups was observed for cycling to school (67.2% versus 65.7%; *p* = 0.715). In addition, adolescent perceptions of having an excessively heavy school bag to carry differed by the mode of transport to school (Figure 2B,D). Among ATS users, 30.9% of adolescents agreed that they carry too much to walk to school (Figure 2B) and 47.9% perceived they carry too much to cycle to school (Figure 2D), compared to 55.9% to 67.7% of combined transport users and 69.2% to 72.8% of motorized transport users. Adolescents’ perception that they carry too much to walk to school was negatively related to the frequency of walking to school and positively related to the frequency of being driven to school (Figure 3). These data are not presented for cycling due to very low rates of cycling to school in the study location (14 adolescents (2%) in this study sample).

### 3.5. Actual School Bag Weights

The average measured school bag weight was 5.6 ± 2.1 kg (range: 0.8–13.3 kg), representing 9.3 ± 3.9% of adolescents’ body weight (range: 1.4%–29.3%) (Table 2). School bag weights were <10% of body weight for 62.1% of adolescents, 10%–15% for 30.5% of adolescents, and exceeded 15% for 7.4% of adolescents. Both absolute and relative school bag weights were higher for boys compared to girls (Table 2). Absolute school bag weight did not differ by adolescents’ age (r = 0.06, *p* = 0.114), body weight category (underweight/healthy weight/overweight/obese: 6.2 ± 2.3 kg/5.6 ± 2.1 kg/5.6 ± 2.0 kg/5.1 ± 2.2 kg; *p* = 0.229), or mode of transport to school (active/motorized/combined: 5.4 ± 2.1 kg/5.7 ± 2.1 kg/5.5 ± 2.0 kg; *p* = 0.432). However, relative school bag weight (as a proportion of body weight) was negatively correlated with age (r = −0.19, *p* < 0.001) and significantly differed across weight status categories (Table 2). Underweight adolescents carried school bags that were, on average, 15% of their body weight. There were no significant differences between relative school bag weights by mode of transport to school (Table 2). Absolute and relative school bag weights did not correlate with distance to school (r = 0.07, *p* = 0.06, and r < 0.01, *p* = 0.053, respectively).

## 4. Discussion

The key findings from this study are as follows: (a) on average, absolute school bag weight among New Zealand adolescents was 5.6 kg (equivalent to 9.3% of adolescents’ body weight), with significant gender and body weight category differences; (b) more than half of parents and adolescents perceived that the adolescents’ school bags were too heavy for walking or cycling to school; and (c) adolescent and parental perceptions differed by adolescents’ mode of transport to school, although the actual school bag weights did not differ by mode of transport. Taken together, these findings suggest that school bag weight is a significant public health issue, particularly for boys and underweight adolescents. In addition, perceived rather than actual school bag weights represent one barrier to ATS for adolescents and their parents and should be considered in future ATS interventions.

In the present study, the average absolute school bag weight among New Zealand adolescents was 5.6 kg. This is less than previously reported in another New Zealand study (6.6 kg) [33] but similar to the findings reported for Australian adolescents (5.3 kg) [6]. In contrast, reported school bag weights were notably lighter for adolescents from Iran (2.8 kg) [2], Uganda (3.8 kg) [7], and Denmark (4.0 kg) [8]. In the present study, adolescents’ school bags were, on average, 9.3% of their body weight. Previous studies reported a range of relative school bag weights in New Zealand (13.2% and 10.3% in younger and older adolescents, respectively) [33] and Iranian adolescents (7.1%) [2]. Currently there is no global agreement on the cutoff point for a school bag weight limit for children and adolescents. While most of the current guidelines suggest a maximum bag weight between 10% and 15% of body weight, recommended weights can be as low as 5% and as high as 20% of a child’s body weight [9,10,11]. However, maximum school bag weight recommendations are generally not supported by the literature [34]. In addition, some authors suggest that school bag weight limits should take into account age (i.e., different limits for children of different age groups) [1] and gender (a lower limit for girls than boys) [2]. However, not all cross-sectional studies have found an association between school bag weight and musculoskeletal complaints in children and adolescents [35], and long-term follow-up studies are necessary [34].

Although absolute school bag weights were not significantly associated with age, relative school bag weights were weakly but significantly negatively correlated with age in the present study. Previous studies reported higher relative school bag weights for younger versus older adolescents [2,33,36]. Although some studies showed that younger children who carried heavier school bags were at increased risk for developing back pain compared to older children [1], the recent systematic review did not find an association between school bag use and risk of back pain in children and adolescents [5]. 

In the present study, measured school bags were heavier for adolescent boys than girls. Inconsistent findings have been reported in the literature, with lighter school bags observed among girls versus boys in Greece [37] and no gender difference in relative school bag weight reported for New Zealand adolescents [33]. However, there is evidence suggesting that girls are more likely to report pain and fatigue symptoms experienced by carrying school bags [2,6,37,38].

To our knowledge, this is the first study to examine adolescents’ actual school bag weights and perceptions across different body weight categories. In the present study, underweight adolescents carried school bags that were, on average, 15% of their body weight compared to an average of 7.9% and 5.7% of body weight for overweight and obese adolescents, respectively. Interestingly, compared to underweight and healthy-weight adolescents, a higher proportion of overweight and obese adolescents perceived that they had too much to carry to walk to school. Although a recent systematic review did not find evidence that school bag use increases the risk of back pain in children and adolescents [5], our findings have significant public health implications and suggest that school bag weight may be a particular issue for underweight adolescents. Therefore, future interventions for reducing school bag weight should focus on underweight adolescents. In addition, these results demonstrate that school bag weight may represent a greater perceived barrier to walking to school among overweight and obese adolescents compared to their healthy-weight and underweight peers. Future studies should examine whether underweight adolescents are more likely to develop and report musculoskeletal complaints and whether such musculoskeletal problems are related to the weight of their school bag. 

In the present study, absolute and relative school bag weights did not differ by adolescents’ mode of transport to school. However, adolescent and parental perceptions that school bags were too heavy to walk or cycle to school differed by adolescents’ mode of transport to school. A lower proportion of adolescents and their parents who used ATS perceived that their school bags were too heavy to walk or cycle to school compared to their counterparts who used only motorized or combined active and motorized transport. Few previous studies examined children’s perception of their school bag weight [27,28] and the relationship between school bag characteristics and mode of transport to school [1,2]. Two US studies conducted with children aged 11 to 14 years found that 37% to 65% of children considered their school bags to be “heavy” and only 5% to 6% regarded their school bags as “light.” One study reported a significant influence of the duration of carrying a school bag on the choice of transportation to school [2]. Another study reported that primary school children living farther away from school carried fewer books in their school bags compared to others [1]. A study of Iranian adolescents (age 12–14 years) found no difference in time spent carrying a school bag and mode of transport to and from school by gender or year level [2]. Our study is the first to explore the relationship between adolescents’ and parents’ perceptions of how much the adolescents carry to school and their mode of transport. Our findings suggest that adolescent and parental perceptions of school bag weight were more important than actual bag weight in terms of affecting adolescents’ school travel behavior.

### 4.1. Implications

We need a better understanding of the reasons for heavy school bags and effective interventions for reducing the weight of school bags. Schools, parents, and adolescents could all play a significant role in determining the content of school bags. Schools could assist in reducing the weight by not requiring “book-heavy” homework and considering the potentially negative consequences of the digital age, where adolescents may be carrying both a laptop and books in their school bags. Parents and teachers could encourage adolescents to consider which items are necessary to carry to school. Although educational interventions with teachers and parents are worthwhile, such interventions are not sufficient [39]. Younger and underweight adolescents are likely to be more affected by heavy school bags, whereas overweight and obese adolescents are likely to perceive their school bags to be too heavy to walk to school. Future recommendations for safe school bag weight limits for adolescents need to take into account both their age and weight status. Future initiatives to promote ATS, and particularly cycling, among adolescents should also consider interventions to reduce the weight of school bags. However, in addition to perceived and actual school bag weights, other factors, such as distance to school and the availability of safe routes for walking and cycling, also need to be taken into consideration in future ATS initiatives. Such initiatives should also include educating parents about the benefits of ATS and dropping off adolescents farther away from school when ATS only is not feasible due to distance and/or unsafe routes.

### 4.2. Strengths and Limitations

Study strengths include a large sample size of both parents and adolescents, data on both parental and adolescent perceptions of the weight of school bags as a barrier to walking and cycling to school, measured school bag weights, exclusion of participants with partial school bag weights, and school bag weight analysis by weight status based on measured height and weight of adolescents. Study limitations include the cross-sectional design, exclusion of a large number of participants due to partial school bag weight, school bag weight measurement on one day only, lack of data on mode of bag carrying (i.e., on one or both shoulders) and duration of carrying school bag, and lack of data on musculoskeletal symptoms.

## 5. Conclusions

The findings from this study have significant implications for public health and school policies. We need a better understanding of the reasons for heavy school bags and effective interventions for reducing their weight. Schools, parents, and adolescents could all play a significant role in determining the content of school bags. Schools could assist in reducing the weight by not requiring “book-heavy” homework and considering the potentially negative consequences of the digital age, where adolescents may be carrying both a laptop and books in their school bags. Parents and teachers could encourage adolescents to consider which items are necessary to carry to school. Younger and underweight adolescents are likely to be more affected by heavy school bags, whereas overweight and obese adolescents are likely to perceive their school bags to be too heavy to walk to school. Future recommendations for safe school bag weight limits for adolescents need to take into account both adolescents’ age and weight status. Future initiatives to promote ATS, and particularly cycling, among adolescents should also consider interventions to reduce the weight of school bags.

## Figures and Tables

**Table 1:** Sociodemographic characteristics of the study participants.

	Adolescents (n = 682)	Parents (n = 331)
**Age (years)**	15.1 ± 1.4	47.4 ± 5.1
**Gender (n (%))**		
Male	391 (57.3)	77 (23.3)
Female	291 (42.7)	254 (76.7)
**Ethnicity (n (%))**		
New Zealand European	510 (74.9)	250 (75.5)
Māori	74 (10.9)	20 (6.0)
Other	95 (14.0)	61 (18.4)
**Transport to school (n (%))**		
Active transport	167 (24.5)	–
Motorized transport	412 (60.5)	–
Combined active and motorized transport	102 (15.0)	–

**Table 2:** Measured school bag weights.

	Adolescents: Total sample (n = 682)	*p*-Value	Boys (n = 391)	Girls (n = 291)	*p*-Value for Boys vs. Girls
Absolute school bag weight (kg)	5.6 ± 2.1		6.0 ± 2.2	4.9 ± 1.6	<0.001
Relative school bag weight (% of body weight)	(n = 663)		(n = 388)	(n = 279)	
Relative school bag weight, average	9.3 ± 3.9		9.7 ± 4.1	8.7 ± 3.5	0.005
Relative school bag weight by school bag weight threshold category (n (%))					
<10% of body weight	412 (62.1)		226 (58.2)	186 (67.6)	
10.0% to 14.9% of body weight	202 (30.5)		127 (32.7)	75 (27.3)	
≥15% of body weight	49 (7.4)		35 (9.0)	14 (5.1)	0.027
Relative school bag weight by adolescent weight status (%)	(n = 660)		(n = 386)	(n = 274)	
Underweight	15.0 ± 5.2		14.5 ± 6.0	15.8 ± 3.6	
Healthy weight	9.7 ± 3.7		10.2 ± 3.9	9.1 ± 3.2	
Overweight	7.9 ± 2.8		8.1 ± 2.9	7.5 ± 2.7	
Obese	5.7 ± 2.5	<0.001	6.2 ± 2.8	5.9 ± 2.0	0.863
Relative school bag weight by mode of transport to school (%)	(n = 662)		(n = 387)	(n = 275)	
Active transport only	9.3 ± 3.8		9.9 ± 3.9	8.6 ± 3.5	
Motorized transport only	9.4 ± 4.0		9.7 ± 4.2	8.8 ± 3.3	
Combined active and motorized transport	9.1 ± 3.8	0.866	9.5 ± 4.3	8.9 ± 3.6	0.189

## References

[1] Adeyemi A.J., Rohani J.M., Rai M.R. (2014). Back pain arising from schoolbag usage among primary schoolchildren. Int. J. Ind. Ergon..

[2] Dianat I., Sorkhi N., Pourhossein A., Alipour A., Asghari-Jafarabadi M. (2014). Neck, shoulder and low back pain in secondary schoolchildren in relation to schoolbag carriage: Should the recommended weight limits be gender-specific?. Appl. Ergon..

[3] Mikkelsson M., Salminen J.J., Kautiainen H. (1997). Non-specific musculoskeletal pain in preadolescents. Prevalence and 1-year persistence. Pain.

[4] Perquin C.W., Hazebroek-Kampschreur A.A., Hunfeld J.A., Bohnen A.M., van Suijlekom-Smit L.W., Passchier J., van der Wouden J.C. (2000). Pain in children and adolescents: A common experience. Pain.

[5] Yamato T.P., Maher C.G., Traeger A.C., Wiliams C.M., Kamper S.J. (2018). Do schoolbags cause back pain in children and adolescents? A systematic review. Br. J. Sports Med..

[6] Grimmer K., Williams M. (2000). Gender-age environmental associates of adolescent low back pain. Appl. Ergon..

[7] Mwaka E.S., Munabi I.G., Buwembo W., Kukkiriza J., Ochieng J. (2014). Musculoskeletal pain and school bag use: A cross-sectional study among Ugandan pupils. BMC Res. Notes.

[8] Skoffer B.B. (2007). Low back pain in 15- to 16-year-old children in relation to school furniture and carrying of the school bag. Spine.

[9] The American Academic of Pediatrics Back to School Tips from the American Academy of Pediatrics 2017. https://www.aap.org/en-us/about-the-aap/aap-press-room/news-features-and-safety-tips/pages/back-to-school-tips.aspx.

[10] Rateau M.R. (2004). Use of backpacks in children and adolescents. A potential contributor of back pain. Orthop. Nurs..

[11] Moore M.J., White G.L., Moore D.L. (2007). Association of relative backpack weight with reported pain, pain sites, medical utilization, and lost school time in children and adolescents. J. Sch. Health.

[12] Tremblay M.S., LeBlanc A.G., Kho M.E., Saunders T.J., Larouche R., Colley R.C., Goldfield G., Connor Gorber S. (2011). Systematic review of sedentary behaviour and health indicators in school-aged children and youth. Int. J. Behav. Nutr. Phys. Act..

[13] Corder K., Sharp S.J., Atkin A.J., Griffin S.J., Jones A.P., Ekelund U., van Sluijs E.M. (2015). Change in objectively measured physical activity during the transition to adolescence. Br. J. Sports Med..

[14] Mandic S., García Bengoechea E., Coppell K.J., Spence J.C. (2017). Clustering of (un)healthy behaviors in adolescents from Dunedin, New Zealand. Am. J. Health Behav..

[15] Maddison R., Marsh S., Hinckson E., Duncan S., Mandic S., Taylor R., Smith M. (2016). Results from the New Zealand’s 2016 Report Card on Physical Activity for Children and Youth. J. Phys. Act. Health.

[16] Faulkner G.E.J., Buliung R.N., Flora P.K., Fusco C. (2009). Active school transport, physical activity levels and body weight of children and youth: A systematic review. Prev. Med..

[17] Mendoza J.A., Watson K., Nguyen N., Cerin E., Baranowski T., Nicklas T.A. (2011). Active commuting to school and association with physical activity and adiposity among US youth. J. Phys. Act. Health.

[18] Voss C., Sandercock G. (2010). Aerobic fitness and mode of travel to school in English schoolchildren. Med. Sci. Sports Exerc..

[19] Daly-Smith A.J.W., McKenna J., Radley D., Long J. (2011). The impact of additional weekdays of active commuting on children achieving a criterion of 300+ minutes of moderate-to-vigorous physical activity. Health Ed. J..

[20] Guthold R., Cowan M.J., Autenrieth C.S., Kann L., Riley L.M. (2010). Physical activity and sedentary behavior among schoolchildren: A 34-country comparison. J. Pediatr..

[21] Tremblay M.S., Barnes J.D., Gonzalez S.A., Katzmarzyk P.T., Onywera V.O., Reilly J.J., Tomkinson G.R., Global Matrix 2.0 Research Team (2016). Global Matrix 2.0: Report card grades on the physical activity of children and youth comparing 38 countries. J. Phys. Act. Health.

[22] Gray C.E., Larouche R., Barnes J.D., Colley R.C., Bonne J.C., Arthur M., Cameron C., Chaput J.P., Faulkner G., Janssen I. (2014). Are we driving our kids to unhealthy habits? Results of the Active Healthy Kids Canada 2013 report card on physical activity for children and youth. Int. J. Environ. Res. Public Health.

[23] Ministry of Transport (2015). 25 Years of New Zealand Travel: New Zealand Household Travel 1989–2014.

[24] Mandic S., Leon de la Barra S., Garcia Bengoechea E., Stevens E., Flaherty C., Moore A., Middlemiss M., Williams J., Skidmore P. (2015). Personal, social and environmental correlates of active transport to school among adolescents in Otago, New Zealand. J. Sci. Med. Sport.

[25] Hopkins D., Mandic S. (2017). Perceptions of cycling amongst high school students and their parents. Int. J. Sustain. Transp..

[26] Mandic S., Hopkins D., García Bengoechea E., Flaherty C., Williams J., Sloane L., Moore A., Spence J.C. (2017). Adolescents’ perceptions of cycling versus walking to school: Understanding the New Zealand context. J. Transp. Health.

[27] Pascoe D.D., Pascoe D.E., Wang Y.T., Shim D.M., Kim C.K. (1997). Influence of carrying book bags on gait cycle and posture of youths. Ergonomics.

[28] Goodgold S., Corcoran M., Gamache D., Gillis J., Guerin J., Coyle J.Q. (2002). Backpack use in children. Pediatr. Phys. Ther..

[29] Mandic S., Mountfort A., Hopkins D., Flaherty C., Williams J., Brook E., Wilson G., Moore A. (2015). Built Environment and Active Transport to School (BEATS) Study: Multidisciplinary and multi-sector collaboration for physical activity promotion. Retos.

[30] Mandic S., Williams J., Moore A., Hopkins D., Flaherty C., Wilson G., García Bengoechea E., Spence J.C. (2016). Built Environment and Active Transport to School (BEATS) Study: Protocol for a cross-sectional study. BMJ Open.

[31] Salmond C., Crampton P., Atkinson J. (2007). NZDep2006 Index of Deprivation.

[32] Cole T.J., Bellizzi M.C., Flegal K.M., Dietz W.H. (2000). Establishing a standard definition for child overweight and obesity worldwide: International survey. BMJ.

[33] Whittfield J.K., Legg S.J., Hedderley D.I. (2001). The weight and use of schoolbags in New Zealand secondary schools. Ergonomics.

[34] Dockrell S., Simms C., Blake C. (2013). Schoolbag weight limit: Can it be defined?. J. Sch. Health.

[35] Trevelyan F.C., Legg S.J. (2011). Risk factors associated with back pain in New Zealand school children. Ergonomics.

[36] Aprile I., Di Stasio E., Vincenzi M.T., Arezzo M.F., De Santis F., Mosca R., Briani C., Di Sipio E., Germanotta M., Padua L. (2016). The relationship between back pain and schoolbag use: A cross-sectional study of 5318 Italian students. Spine J..

[37] Kellis E., Emmanouilidou M. (2010). The effects of age and gender on the weight and use of schoolbags. Pediatr. Phys. Ther..

[38] Navuluri N., Navuluri R.B. (2006). Study on the relationship between backpack use and back and neck pain among adolescents. Nurs. Health Sci..

[39] Negrini S., Politano E., Carabalona R., Tartarotti L., Marchetti M.L. (2004). The backpack load in schoolchildren: clinical and social importance, and efficacy of a community-based educational intervention. A prospective controlled cohort study. Eura Medicophys..

